# SARS-CoV-2 and Its Variants in Thrice-Infected Health Workers: A Case Series from an Italian University Hospital

**DOI:** 10.3390/v14112536

**Published:** 2022-11-16

**Authors:** Maria Grazia Lourdes Monaco, Gianluca Spiteri, Gulser Caliskan, Virginia Lotti, Angela Carta, Davide Gibellini, Giuseppe Verlato, Stefano Porru

**Affiliations:** 1 Occupational Medicine Unit, University Hospital of Verona, 37134 Verona, Italy; 2 Section of Epidemiology and Medical Statistics, Department of Diagnostics and Public Health, University of Verona, 37134 Verona, Italy; 3 Section of Microbiology, Department of Diagnostics and Public Health, University of Verona, 37134 Verona, Italy; 4 Section of Occupational Medicine, Department of Diagnostics and Public Health, University of Verona, 37134 Verona, Italy

**Keywords:** SARS-CoV-2, reinfections, multiple infections, thrice-infected, variants of concern, health workers

## Abstract

Background: We described a SARS-CoV-2 thrice-infected case series in health workers (HW) to evaluate patient and virus variants and lineages and collect information on variables associated with multiple infections. Methods: A retrospective analysis of clinical and laboratory characteristics of SARS-CoV-2 thrice-infected individuals was carried out in Verona University Hospital, concurrent with the ORCHESTRA project. Variant analysis was conducted on a subset of available specimens. Results: Twelve HW out of 7368 were thrice infected (0.16%). Symptomatic infections were reported in 63.6%, 54.5% and 72.7% of the first, second and third infections, respectively. Nine subjects were fully vaccinated at the time of the third infection, and five had an additional booster dose. The mean time to second infection was 349.6 days (95% CI, 138–443); the mean interval between the second and third infection was 223.5 days (95% CI, 108–530) (*p* = 0.032). In three cases, the second and third infections were caused by the Omicron variant, but different lineages were detected when the second vs third infections were sequenced. Conclusions: This case series confirms evidence of multiple reinfections with SARS-CoV-2, even from the same variant, in vaccinated HW. These results reinforce the need for continued infection-specific prevention measures in previously infected and reinfected HW.

## 1. Introduction

SARS-CoV-2 reinfections were reported in mid-2020, raising concerns about natural immunity [1]. The onset of SARS-CoV-2 reinfection represents an obstacle in handling the pandemic since it defies the herd immunity concept and control measures [2]. It is reported that SARS-CoV-2 can reinfect fully vaccinated individuals. The frequency of reinfection was not determined among unvaccinated, partially vaccinated, and fully vaccinated individuals, even though the vaccination reduces the severity of infection [3]. It is noteworthy that a key role in reinfection is played by SARS-CoV-2 genome mutations, thus inducing the appearance of new variants with different clinical characteristics [4]. A deeper understanding of viral and immunologic features of SARS-CoV-2 reinfections may help define reliable correlates of immunity [5].

In this report, we evaluated individual clinical variables, virus variants, and lineages in a series of thrice-infected health workers (HW).

## 2. Materials and Methods

A retrospective cohort of SARS-CoV-2 reinfection cases was carried out at the University Hospital of Verona from 24 February to 10 August 2020, 2022, among 7638 HW, within a dynamic cohort [6] included in the ORCHESTRA project, a 15 countries multi-centre study.

Reinfections were identified by screening and contact tracing, including those that occurred >90 days after the prior infection and if new COVID-19 symptoms began after the resolution of prior symptoms [7]. Only HW having multiple SARS-CoV-2 infections with thrice-positive swabs were included. Patient, infection, and virus characteristics were collected. 

SARS-CoV-2 genome detection was performed on nasopharyngeal swabs via RT-PCRs. Samples previously tested positive were analysed to assess the SARS-CoV-2 variant by Novaplex™ SARS-CoV-2 Variants VII Assay (Seegene, Seoul, South Korea). 

## 3. Results

Twelve HW thrice SARS-CoV-2 infected (8 males and 4 females) were detected, with a mean (±SD) age of 44 y (±9.4). Clinical data were available for 11 HW: two had allergies, one had hypertension. BMI was in the healthy weight range for all HW. Moreover, 14/33 infections were occupational, 8 originated from household contacts. The source was unknown in 11. 

At the time of 33 infections, 10 HW were not vaccinated, 10 fully vaccinated, 7 up to date boosters, and 6 partially vaccinated. As regards the vaccine type, 11/12 HW received BNT162b2, while 1 HW received only two doses of mRNA-1273. 30% of reinfections were considered breakthrough infections.

The mean interval was 349.6 days (95% CI, 138–443) between the first and second infections, while the mean interval between the second and third infection was 223.5 days (95% CI, 108–530) (*p* = 0.032)

Table 1 shows HW characteristics and infection details, including the variant analysis for each individual.

**Figure 1:**
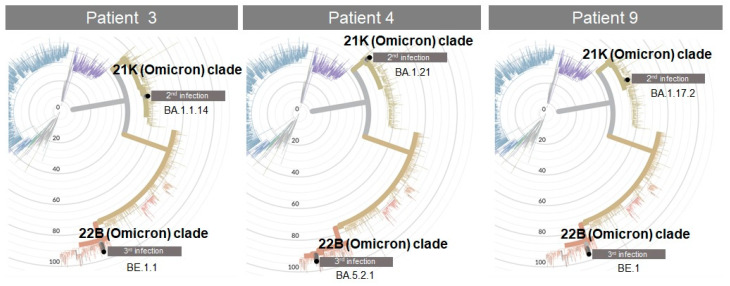
Phylogenetic tree displaying sequenced SARS-CoV-2 strains.

Figure 1 illustrates a radial phylogenetic tree of sequenced SARS-CoV-2 strains for the analysed patients, according to the designated clades of the virus. The tree was generated by the Nextclade site comparing the two different Omicron lineages of the second and third infection of patients 3, 4, and 9, displaying the phylogenetic distance between the two samples. In all cases, an Omicron variant was detected.

Since the Novaplex kit is not able to differentiate the Omicron variant BA.4 and BA.5 with respect to the BA.1 and BA.2, we performed sequencing analysis by NGS procedure in these samples. The results indicated that the lineages were different. In fact, all second infections were classified in the 21K Omicron variant, during the third infection in the 22B Omicron variant (Figure 1, Table 1) in the BA.5 lineage.

**Figure 2:**
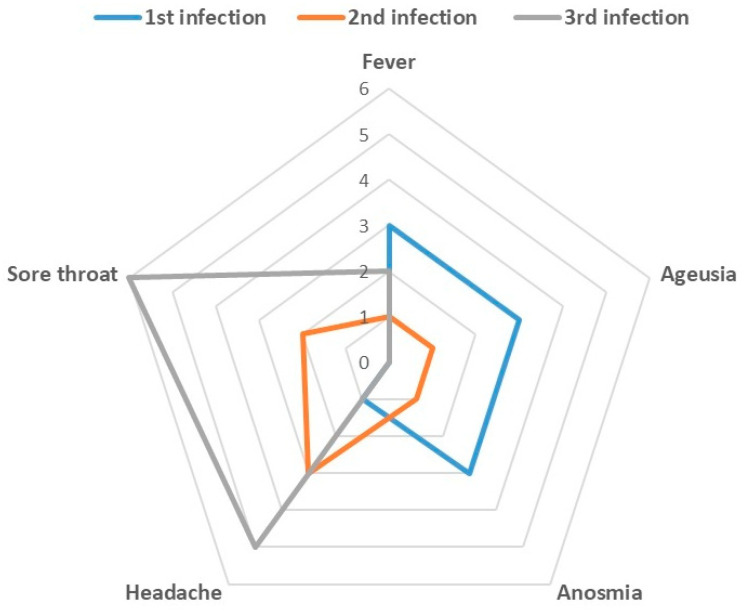
Description of symptoms characteristics by infections time and vaccination status.

**Figure 3:**
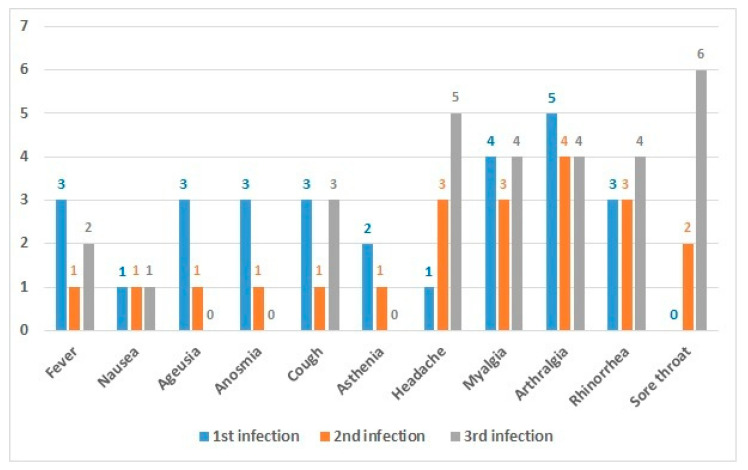
Description of symptoms characteristics by infections time and vaccination status.

Table 2 displays symptom categories (no symptoms, minor, major, hospitalisation) and vaccination status in first, second, and third infections, while Figure 2 and Figure 3 detail the type of symptoms. The median value (IQ25–75) of symptoms duration among 1st, 2nd, and 3rd infections was 4 days (0-6), 0 (0-4) and 3 (1, 5-3,5), respectively. 

Table 3 reports the cycle threshold (Ct) values for the analysed specimens.

## 4. Discussion

Very few data are available on SARS-CoV-2 thrice infected. To our knowledge, only another study with a similar number of cases of HW infected more than twice is available [9] in a tenfold larger population. Our data could be linked to HW periodical SARS-CoV-2 screening, but a similar outcome might occur in the general population. 

As reported by Swift et al., multiple infections can also occur in young, immunocompetent and vaccinated individuals, and comorbidities do not seem to play a key role. Indeed, among the three subjects who reported comorbidities in our study (25%), two had mild allergic respiratory diseases, and one had arterial hypertension on drug therapy. Neither in this study nor in Swift’s was affected by immunosuppression. It is therefore probable that one or multiple previous infections, as well as vaccination and comorbidities, influence the disease’s severity rather than the risk of infection, in particular after the onset of variants of concern (VOC). 

Even a young age does not seem to be protective against the risk of multiple reinfections. Both our study and Swift’s showed a low median age (46 and 27, respectively) [9]. 

Regarding the interval between the infections, we found that the second and third infections had a shorter lag time than that between the first and second infections. These results align with those highlighted by Swift et al., and they seem to suggest that VOC also have a major impact on this aspect.

Our data show that screening testing maintains a primary role in the prevention of the infection spreading, especially in high-risk categories and previously infected subjects. Indeed, fourteen out of 33 (42.4%) infections were detected through periodic testing. Similar results (9/33; 27.3%) were found by Swift et al. (also including pre- and post-travel screening) [9]. 

Although the small number of infections does enable definitive conclusions, this study has some strengths. In many samples, it was possible to identify the SARS-CoV-2 variant related to infection and Ct values. This information increased the specificity of our definition of reinfection. Moreover, clinical data and vaccination status were collected, enabling a better description of infections.

## 5. Conclusions

This study shows multiple SARS-CoV-2 reinfections also in vaccinated HW; interestingly, three patients showed Omicron variant both in the second and third infection, but different lineages were detected.

The continuous infection-specific prevention measures and targeted screening programmes with swabs still represent valuable infection control procedures, especially in at-risk populations such as HW.

## Figures and Tables

**Table 1:** Description of 12 HW and multiple SARS-CoV-2 infections with variants lineages.

No	Workers’ Characteristics (Age-yo-/Sex/Job Title/ Vaccination Dates)	Infections Details (Date, Lag in-Days-, Variant, Lineage, Cause of Swab Testing, Type of Contact)		
		1st infection	2nd infection	3rd infection
**1**	46	21/03/2020	25/01/2021	09/07/2022
	Female	28	17	7
	nurse	Wuhan *	Delta **	BA.1 **
	1st dose: 07/05/2021	Symptoms onset	Strict contact	Strict contact
	2nd dose: 22/12/2021	Occupational	Occupational	Relative/friend
**2**	30	07/04/2020	10/06/2021	12/02/2022
	male	15	7	7
	physician	Wuhan *	B.1.1.7 *	BA.1 **
	1st dose: 07/01/2021	Screening	Screening	Screening
	2nd dose: 28/01/2021 3rd dose: 09/11/2021	Unknown	Unknown	Unknown
**3**	33	14/03/2020	12/02/2022	10/07/2022
	female	31	10	10
	nurse	Wuhan *	BA.1 ** (BA.1.1.14 ^†^)	BA.1 ** (BE.1.1 ^†^)
	1st dose: 07/05/2021	Strict contact	Screening	Symptoms onset
	2nd dose: 28/05/2021	Occupational	Unknown	Occupational
**4**	46	29/03/2021	16/02/2022	22/07/2022
	female	7	15	7
	nurse	B.1.1.7 *	BA.1 ** (BA.1.21 ^†^)	BA.1 ** (BA.5.2.1 ^†^)
	1st dose: 11/02/2022	Screening	Symptoms onset	Symptoms onset
	2nd dose:03/06/2022	Unknown	Relative/friend	Relative/friend
**5**	49	17/12/2020	04/05/2021	10/01/2022
	male	11	7	7
	physician	Wuhan *	B.1.1.7 *	Omicron *
	1st dose: 05/01/2021	Screening	Screening	Screening
	2nd dose:26/01/2021 3rd dose: 09/11/2021	Unknown	Unknown	Unknown
**6**	40	19/07/2021	01/01/2022	04/08/2022
	male	11	18	7
	nurse	B.1.617.2 *	BA.1 **	BA.5 *
	1st dose: 11/02/2021	Screening	Symptoms onset	Symptoms onset
	2nd dose: 04/03/2021 3rd dose:13/12/2021	Relative/friend	Occupational	Occupational
**7**	48	27/04/2021	16/04/2022	02/08/2022
	female	11	7	7
	nurse	Alfa **	BA.2 **	BA.1 **
	1st dose: 06/01/2021	Strict contact	Screening	Symptoms onset
	2nd dose: 27/01/2021 3rd dose:09/12/2021	Relative/friend	Occupational	Relative/friend
**8**	56	27/10/2020	13/01/2022	18/05/2022
	female	34	7	7
	nurse	Wuhan *	BA.1 **	BA.2 **
	1st dose: 01/09/2021	Strict contact	Strict contact	Strict contact
		Occupational	Occupational	Occupational
**9**	42	13/11/2020	24/01/2022	29/06/2022
	female	10	7	7
	other HW	Wuhan *	BA.1 ** (BA.1.17.2 ^†^)	BA.1 ** (BE.1 ^†^)
	1st dose: 11/10/2021	Symptoms onset	Screening	Screening
		Occupational	Unknown	Unknown
**10**	56	10/11/2020	10/06/2021	13/06/2022
	female	10	7	7
	nurse	Wuhan *	B.1.1.7 *	BA.2 *
	1st dose: 16/03/2021	Strict contact	Screening	Strict contact
	2nd dose:09/12/2021	Occupational	Unknown	Relative/friend
**11**	30	31/12/2020	16/12/2021	21/06/2022
	male	16	7	7
	nurse	Wuhan *	Delta **	BA.1 **
	1st dose: 16/02/2021	N/A	N/A	N/A
	2nd dose: 10/03/2021 3rd dose:14/12/2021	N/A	N/A	N/A
**12**	54	05/02/2021	03/01/2022	13/07/2022
	female	10	7	9
	nurse	B.1.1.7 *	Omicron *	BA.1 **
	1st dose: 05/02/2021	Strict contact	Screening	Symptoms onset
	2nd dose: 26/02/2021 3rd dose:16/12/2021	Occupational	Relative/friend	Occupational

* Not laboratory identified but assumed based on the loco-regional epidemiological data on the dominant variant [8]. ** Samples previously tested positive were analysed to assess the SARS-CoV-2 variant by Novaplex™ SARS-CoV-2 Variants VII Assay (Seegene, Seoul, South Korea), enabling distinction between Alfa, Beta/Gamma, Delta, and Omicron BA.1 and BA.2 variants of concern, following manufacturer’s instructions. ^†^ SARS-CoV-2 subvariants obtained by RNA sequencing analysis.

**Table 2:** Symptoms categories and vaccination status of HW thrice infected.

	1st infection											
**HWs no**	**1**	**2**	**3**	**4**	**5**	**6**	**7**	**8**	**9**	**10**	**11**	**12**
No symptoms		U		U	U	F					N/A	
Minor symptoms	U		U				F		U	U	N/A	U
Major symptoms								U			N/A	
Hospitalisation											N/A	
	**2nd infection**											
**HWs no**	**1**	**2**	**3**	**4**	**5**	**6**	**7**	**8**	**9**	**10**	**11**	**12**
No symptoms	U		F		F				P	P	N/A	
Minor symptoms		F		P		B	B	P			N/A	F
Major symptoms											N/A	
Hospitalisation											N/A	
	**3rd infection**											
**HWs no**	**1**	**2**	**3**	**4**	**5**	**6**	**7**	**8**	**9**	**10**	**11**	**12**
No symptoms		B			B				P		N/A	
Minor symptoms	F		F	F		B	B			F	N/A	B
Major symptoms								P			N/A	
Hospitalisation											N/A	

U = unvaccinated; P = partially vaccinated; F = fully vaccinated; B = vaccinated with booster dose.

**Table 3:** Cycle threshold value at 1st, 2nd and 3rd positivity for each HW enrolled in the case series.

No	1st Positivity			2nd Positivity			3rd Positivity		
	**Allplex™ SARS-CoV-2 Assay (Seegene)**								
	**E Gene**	**S Gene**	**N Gene**	**E Gene**	**S Gene**	**N Gene**	**E Gene**	**S Gene**	**N Gene**
**1**	19.39	21.18	22.18	32.64	32.23	30.28	37.32		37.38
**2**							29.95	31.09	28.49
**3**	17.63	18.98	21.50	26.64	27.19	24.06	28.34	30.52	27.70
**4**				32.76	34.11	31.57	28.06	28.08	26.75
**5**							32.75	32.69	34.03
**6**	38.13		38.51	37.60	35.92	34.30		21.16	22.13
**7**	26.85	31.28	33.57				22.41	23.66	21.34
**8**				23.21	23.45	20.88	20.34	20.91	19.69
**9**				25.85	28.64	24.87		37.00	37.00
**11**	32.96	34.25	29.77	36.70		39.10			
**12**				34.39	36.38	34.62		22.13	20.22
	**EurobioPlex SARS-CoV-2 Multiplex (Eurobio Scientific)**								
	**RdRp gene** **(Target 1)**	**RdRp gene** **(Target 2)**	**N gene**						
**4**	35.00	35.00	35.00						
	**Simplexa™ COVID-19 Direct Kit (DiaSorin Molecular)**								
				**Orf1ab**	**S gene**		**Orf1ab**	**S gene**	
**7**				31.80	29.40				
**11**							20.00	19.00	
	**TaqPath™ COVID-19 RT-PCR Kit (Applied Biosystem)**								
	**Orf1ab**	**S gene**	**N gene**						
**10**	20.70	20.60	22.20						

## Data Availability

The datasets generated during the current study are not publicly available, because they contain sensitive data to be treated under data protection laws and regulations. Appropriate forms of data sharing can be arranged after a reasonable request to the last author.
